# Welcome, *PNAS Nexus*!

**DOI:** 10.1093/pnasnexus/pgab002

**Published:** 2022-03-02

**Authors:** Marcia McNutt

**Affiliations:** President, National Academy of Sciences, 500 Fifth Street, NW, Washington, DC 20001, USA

The launch of a new journal is a very exciting time for any organization. The arrival of the first issue is almost as eagerly awaited as the birth of a new child, although the planning may consume much more than 9 months. *PNAS Nexus* is the first fully open-access, peer-reviewed research journal to be published by the National Academy of Sciences (NAS) and the first to involve a partnership with Oxford University Press, the National Academy of Engineering, and the National Academy of Medicine.

However, some readers may be skeptical of the need for this new journal. After all, the internet is awash with research publications, some of questionable purpose and quality, and no doubt a few with no other rationale than to extract funds from authors. Let me assure those who hesitate to embrace this new journal that the need for it is clear.

**Figure 1:**
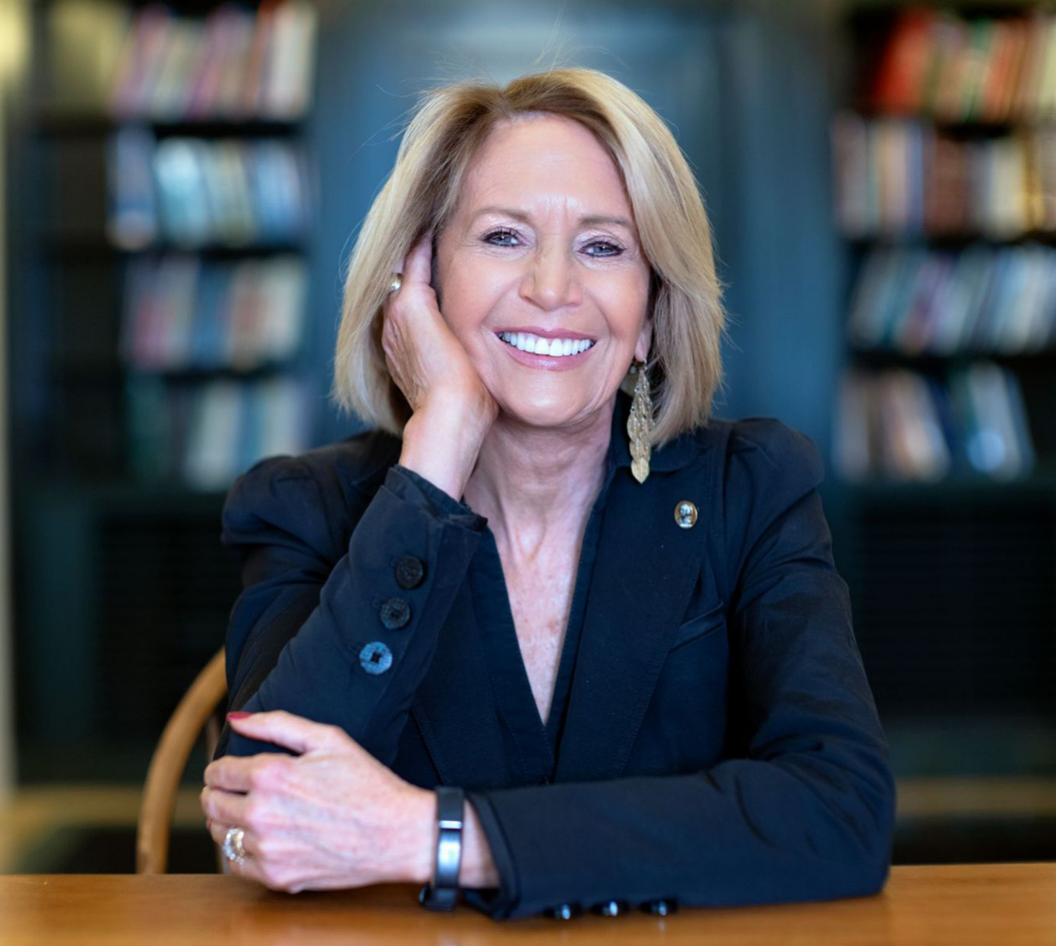
Marcia McNutt

First, *Nexus* builds on the reputation of PNAS for high quality in the selection and publication of cutting-edge, high-impact research. The editorial team draws from distinguished members of the three National Academies—the NAS, the National Academy of Engineering, and the National Academy of Medicine—in addition to other experts, who will oversee a rigorous peer-review system. However, unlike PNAS, *Nexus* will not focus primarily on basic scientific research but will broadly encompass engineering and medical research, with a particular emphasis on the intersection (or “nexus”) of these three domains. Some of the most exciting discoveries are now being advanced through the use of convergence: “the integration of engineering, physical sciences, computation, and life sciences—with profound benefits for medicine and health, energy, and environment” (1). Despite the success of adopting convergence to address the challenges of our time, there is a dearth of high-quality publishing outlets that encompass the full breadth of the disciplines contributing to the research. *Nexus* will provide such a venue without the risk of de-emphasizing the particular contributions from science, engineering, or medical research.

Next, *Nexus* provides a gold Open Access option for authors who seek broad dissemination of their results from a high-quality journal. For convergence research, the journal reaches scientific, engineering, and medical researchers as well as those interested in applications to society's challenges. While many journals, including PNAS, offer one or more open-access publishing licenses, some funders with open access mandates do not allow research they support to appear in “hybrid” journals. *Nexus* meets all funder mandates.

A final observation is that the three US National Academies have been increasingly working together to confront the growing challenges to a just, resilient, and sustainable future for humankind on this planet. An excellent recent example has been our combined efforts to address the broad impacts of the coronavirus disease 2019 (COVID-19) pandemic: to recommend a protocol for vaccine allocation, to confront supply chain issues, and to counter misinformation and vaccine hesitancy. It is high time that we published a journal that is able to combine the full scope of work that we do both individually and together as academies.

While the launch of a new journal may seem like a risky endeavor for an organization as cautious as the NAS, there is proof that this approach holds promise. Years ago when I was editor-in-chief of *Science* magazine, we launched a fully open access sister journal, *Science Advances*. Like *Nexus*, *Science Advances* was broader in scope than its established sibling. Quickly this new journal established its own reputation and following. Despite only publishing a relatively small number of papers in its first partial year, three of its papers made the list of the Altmetric Top 100 that year. Its debut impact factor was the highest of any open access journal at launch, and it has steadily risen over the years. *Science* itself as a journal did not suffer with the addition of the new title. In fact, it might have even benefitted from authors seeing a path to have their papers sequentially considered for publication in two high-quality outlets without reformatting, given the high rejection rate for *Science*.

Inaugural Editor-in-Chief of *Nexus*, Karen Nelson, brings fresh ideas and a broad perspective to this new undertaking. Like PNAS, *Nexus* will be under the immediate oversight of the NAS Committee on Publications and indirectly under the purview of the NAS Council. I wish Dr. Nelson and the entire *Nexus* editorial team much success with this new undertaking and thank them for their service to the broad dissemination of cutting-edge science.
